# The Exploration of Disturbance of Capillary and Photoreceptor Communication Networks in Diabetic Retinopathy Through Single‐Cell RNA‐Seq

**DOI:** 10.1111/jcmm.70442

**Published:** 2025-03-03

**Authors:** Ning Wang, Huibo Li, Qinqin Sun, Xuelian Han, Sheng Su

**Affiliations:** ^1^ Eye Hospital, The First Affiliated Hospital Harbin Medical University Harbin Heilongjiang China; ^2^ Department of Hematology, The First Affiliated Hospital Harbin Medical University Harbin Heilongjiang China

**Keywords:** ANGPTL4, cell communication, diabetic retinopathy; endothelial cells, photosensitive cells

## Abstract

This study investigates the differences in ligand–receptor interactions between the communication network of vascular endothelial cells (ECs) and photoreceptor cells (PRCs)in diabetic retinopathy (DR) the mechanism was verified by animal experiments. The GSE209872 data set, including retinal specimens from five Sprague–Dawley rats induced by streptozotocin, was obtained from Gene Expression Omnibus. CM and EC data were extracted individually for reclustering, functional enrichment and trajectory analyses. Cell communication analysis was conducted to investigate the altered signals and significant ligand–receptor interactions. Moreover, novel ligand–receptor interactions were validated using immunofluorescence staining using 2, 4 and 8 weeks DR model; DR was treated with AAV‐shANGPTL4, and retinal function was detected by Haematoxylin and eosin staining (HE), TUNEL and ELISA. The expression of ligand–receptor in DR Retina was detected by qPCR and immunohistochemistry. Nine cell types were determined in DR. Cellular communication results revealed four signalling pathways, including PTN, MK, ANGPTL and CXCL, that were significantly changed in DR. Furthermore, 3 ligand‐receptor pairs (Ptn‐Ncl, Mkd‐Ncl and Angptl4‐Sdc4) were obviously upregulated between ECs and PRCs, the expression of which was verified via immunofluorescence in the DR model. After treatment with AAV‐shANGPTL4, the retinal thickness and average density of RGCs were decreased (*p* < 0.05). TUNEL staining showed that knocking down ANGPTL4 reduced the apoptosis of DR (*p* < 0.05), and VEGF and IGF‐1 expression were downregulated (*p* < 0.01). The expression of ligand–receptors also decreased in the DR Model (*p* < 0.01). The vascular ECs and PRCs demonstrate significant heterogeneities in DR. ANGPTL4 was a decreased ligand–receptor expression and improved retinal function as a potential therapeutic target against DR.

## 1 Introduction

Diabetic retinopathy (DR) is a prevalent complication of diabetes mellitus and remains a significant cause of vision loss and blindness globally [1]. The World Health Organization reported that approximately 33.3% of people with diabetes demonstrated signs of retinopathy, and approximately 10% have vision‐threatening retinopathy [2, 3]. Laser photocoagulation, vitrectomy and intravitreal pharmacotherapeutics are the main therapies for DR, whereas patients with DR at the advanced stage still have a high rate of blindness [4] due to the complex pathological mechanism. Thus, investigating the pathological mechanism of DR would help decrease the rate of blindness.

The blood–retina barrier (BRB) is a specialised barrier that separates blood circulation from the neural tissue of the retina [5]. It consists of two components: the inner BRB (iBRB), which is formed by the endothelial cells (ECs) of the retinal capillaries, and the outer BRB (oBRB), which is formed by the retinal pigment epithelium (RPE) and its tight junctions [6]. ECs in the iBRB regulate the transport of molecules into and out of the retinal tissue [7]. They form tight junctions that restrict the movement of substances between the blood and neural tissue, thereby maintaining retinal homeostasis. These cells control the passage of nutrients, ions and other essential molecules required for normal retinal function while preventing the entry of potentially harmful substances [8]. ECs may become damaged or dysfunctional in DR, a group of disorders characterised by abnormal blood vessel growth in the retina [9]. This increases blood vessel permeability and BRB disruption, enabling the leakage of plasma proteins, lipids and inflammatory cells into the retinal tissue [10]. The compromised BRB contributes to retinal edema, inflammation and retinopathy progression [11]. Moreover, photoreceptor cells (PRCs), located in the outer layer of the retina, capture light and convert it into electrical signals that are transmitted to the brain for visual processing [12]. Their role in the BRB involves maintaining RPE structural integrity and functionality, which forms the outer component of the barrier. Additionally, the disease process affects PRCs [13, 14]. The increased vascular leakage and inflammation related to retinopathy disrupt the normal functioning of the RPE, causing oxidative stress, inflammation and eventual death of PRCs [15]. This is the leading cause of vision loss and impairment [16, 17]. EC and PRC dysfunction in DR and their communication disrupt BRB and disease development and progression [18].

Thus, we obtained the single‐cell sequencing data of DR from Wang et al., who constructed an atlas of the iBRB in the early stage of DR and elucidated the degeneration of its constituent cells and Müller cells and the regulatory association between them. However, this study did not analyse the role of ECs and PRCs in DR and their correlation between them. This study collected ECs and PRCs and focused on the ligand–receptor interactions between them to reveal the association of long‐term hyperglycemia with the communication network between ECs and PRCs in the DR.

## 2 Materials and Methods

### 2.1 Data Source

The single‐cell RNA‐seq dataset GSE209872 was downloaded from the National Center for Biotechnology Information GEO database. This dataset included five Sprague–Dawley rats, among which two rats were regarded as control, namely WT, and the other three rats were utilised to construct the DR models induced by streptozotocin as experimental groups. The retina was obtained after 2 weeks, 4 weeks and 8 weeks (namely DR1, DR2 and DR3), as DR models were constructed successfully. The sequencing library was developed using 10× Genomics platforms, and then double‐terminal sequenced through Illumina NovaSeq 6000 with a read length of 500 bp.

### 2.2 ScRNA‐Seq Analysis

A clustering Seurat object was produced according to all the samples with the Read 10 × () function in the Seurat package (version 4.3.0). The low‐quality cells were excluded (< 350 genes/cell, > 3000 genes/cell, < 3 cells/gene, > 20% mitochondrial genes and 20% ribosomal genes). Subsequently, 35,910 single cells were obtained, consisting of 11,073 cells from normal tissues and 24,837 cells from DR. The FindCluster() function was utilised to perform the clustering of the cells with a resolution of 1.0, which was visualised with the UMAP method. Moreover, the FindAllMarkers() function was carried out to list the markers of the cell clusters. The genes with the threshold of logfc of > 1 and min.pct of 0.25 were regarded as marker genes for certain cell types.

### 2.3 Gene Enrichment Analysis

The R clusterProlifer package (version 4.9.0) was utilised to analyse the Gene Ontology function and Kyoto Encyclopedia of Genes and Genomes (KEGG) pathways involved in certain genesets with the threshold of *p*.adjust of < 0.01. The Gene Set Variation Analysis (GSVA) package (version 1.49.0) in R was utilised to analyse the geneset among different cell types. Further GSVA analysis was conducted to assess the different pathways involved in the differential cell subtypes. Finally, the limma package in R was utilised for determining genesets with the significance of abs (*t* value) of > 10 and FDR adjusted *p* value of < 0.05.

### 2.4 Trajectory Analysis

The Monocle3 package (version 3_1.3.1) [19] was utilised to analyse the differentiation trajectory. The learn_graph() function was used to learn the trajectory of the cells, and order_cells() was then used to arrange the cells following the learned pseudotime. The graph_test() was used to identify the differentially expressed genes (DEGs) in pseudotime with a *q*_value of < 1e‐3. Aggregate_gene_expression() was applied to calculate the aggregation matrix associated with the differential genes and cell types in the quasitemporal trajectory, which was visualised with the R pheatmap package (version 1.0.12).

### 2.5 CellChat Analysis

The CellChat package (version 1.6.1) [20] was used to investigate the interaction of cell–cell contact and secreted signalling.

### 2.6 Validation Using the DR Mice Model

Adult male Sprague–Dawley rats (aged 6–8 weeks, 250–300 g) were employed from Vital River (Beijing, China). Before the experiment, the rats were fed adaptively for 1 week, normally and kept under the light–dark cycle condition. The DR model was successfully constructed using the streptozotocin method based on previous studies [21, 22].

Firstly, 12 SD rats were randomly divided into 4 groups (*n* = 3), control group, 2‐week DR group, 4‐week group and 8‐week group. The DR group was fed a high‐fat diet for 4 weeks, was forbidden to eat for 12 h and was intraperitoneally injected with STZ (40 mg/kg) once. Fasting blood glucose was measured on Days 3 and 7 after injection, and rats with blood glucose above 16.7 mmol/L were used as successful diabetes models. The control group was injected with the same amount of citrate buffer and fed ordinary feed. Three animals were randomly selected in the model group at 2, 4 and 8 weeks. The rats were then anaesthetised using xylazine and ketamine, after which they were sacrificed through cervical dislocation. The eyes were immediately removed, the corneas were incised, and each eye was immersed in 2% paraformaldehyde for 3 h at room temperature. Tissue samples were stored for immunofluorescence assay.

Next, 18 SD rats were randomly divided into three groups (*n* = 6), control group, DR group and AAV‐shANGPTL4 group. Diabetes models were constructed as above. The AAV‐shANGPTL4 group was injected intravitreally with AAV‐shANGPTL4 (Hai Xing Bio, Suzhou, China), an adeno‐associated virus. All animals continued to be fed a regular or high‐fat diet. The experiment ended after 8 weeks of feeding. The serum and retinal tissue of rats were tested.

The Animal Ethics Committee of the First Affiliated Hospital of Harbin Medical University approved each animal experimental procedure.

### 2.7 Immunofluorescence

The fixed retinal paraffin wax was embedded before, and the section was dewaxed and rehydrated. Then 3% H_2_O_2_ was added and incubated at 25°C for 15 min.

Citrate buffer (10 mM, pH = 6) was used for heat‐mediated antigen repair. At room temperature, the slides were sealed with 5% bovine serum albumin for 30 min, and the target antibodies were added and incubated overnight at 4°C. On the second day, Cy3‐labelled goat anti‐Rabbit IgG and Cy3‐labelled goat antimouse IgG were mixed with incubation slices and incubated at room temperature for 2 h. 6‐Diamidino‐2‐phenylindole (DAPI) was incubated for 15 min and then observed and photographed under a fluorescence microscope. Immunofluorescence staining was performed to examine the expression of ligand–receptor interactions, in which red indicated ligands and green indicated receptors.

### 2.8 Haematoxylin and Eosin Staining

To observe the retinopathy of DR rats, haematoxylin and eosin (HE) staining was performed on the retinal tissue of the rats. The paraffin‐embedded retina section was soaked in xylene for 10 min and repeated twice. The tissue samples soaked in xylene were soaked in anhydrous ethanol for 5 min and then soaked in 95%, 85% and 70% ethanol for 5 min each to fully hydrate. Add haematoxylin staining solution for 10 min, differentiate and reverse blue; Continue to add eosin dye for 3 min. After staining, the tissue sections were dehydrated, air‐dried, sealed with neutral gum, observed under a microscope (Tiannuoxiang, Beijing, China) and photographed.

### 2.9 TUNEL Staining

TUNEL staining was used to observe the apoptosis of retinal tissue of DR rats. After routine dewaxing of sections, the retinal tissue was incubated with protease K for 20 min for antigen repair; TUNEL reaction mixture was added, the cell nucleus was labelled by DAPI, the haematoxylin dye was restained for 3 s, the gradient alcohol was dehydrated, and the neutral gum was sealed. TUNEL fluorescence imaging was obtained by fluorescence microscopy.

### 2.10 ELISA Kit Assay

The expression levels of Vascular endothelial growth factor A (VEGFA) and insulin‐like growth factor‐1(IGF‐1) in serum of DR rats were detected by the ELISA kit (Thermo Scientific, Waltham, USA) following the manufacturers' instructions.

### 2.11 QPCR

The mRNA expression levels of ANGPTL4, SDC4, MKD, PTN and NCL were detected by qPCR. The retina of DR Rats was added to Trizol to extract total tissue RNA, and reverse transcription was performed using a reverse transcription kit. Primers were searched and designed on NCBI, and the qPCR primer sequence was as follows: GAPDH‐F:GATTGTTGCCATCAACGACC; GAPDH‐R:GTGCAGGATGCATTGCTGAC; ANGPTL4‐F:TGGTTTGGCACCTGCAGCCATTC; ANGPTL4‐R:TGCTGCCATGGGCTGGATCAAC; SDC4‐F:AAGGTGTCAATGTCCAGCACT; SDC4‐R: GGGCTTTCTTGTAGATGGGTTT; PTN‐F: GTGGAGAATGGCAGTGGAGTGTG; PTN‐R:ACATCTCTGGGTCTTCATGGTTTGC; NCL‐F:CCAAGAAGGAAGACAATGAGG; NCL‐R:TGAGGCAGGAGCAGCAGGAG; MKD‐F:CGAGGCACTTTGGTGTTGAT; MKD‐R: CAGGTGGACCGAGGAGAA. Quanti Fast SYBR Green PCR kit (Solarbio, Beijing, China) was used to conduct Real time PCR reaction by fluorescence quantitative PCR instrument (Bio Rad, California, USA); the procedure was as follows: 95.0°C for 30 s; 40 cycles were performed at 95.0°C for 10 s and 60.0°C for 30 s. The qPCR data were calculated using the 2^−ΔΔCT^ method.

### 2.12 Immunohistochemistry

The expression levels of SDC4 and ANGPTL4 in the retina of DR Rats were observed by immunohistochemical staining. The nuclear part is sky blue, the positive part is brown, indicating the expression of protein. Paraffin sections of retinal tissue samples were placed in an oven at 60°C for 90 min, followed by dewaxing and rehydration with xylene and gradient ethanol. Subsequently, 3% hydrogen peroxide was added to remove endogenous peroxidase blocking solution, and the sections were placed in citric acid repair solution just boiled for antigen repair. After sealing the sections, diluted primary antibody was added, incubated at 4°C overnight, and HRP was added to label sheep antirabbit on the second day, incubated at 37°C for 30 min. Haematoxylin was added for 30 s dyeing, and neutral gum was sealed after dehydration. Observe and photograph under a microscope.

### 2.13 Statistical Analysis

The bioinformatics analyses were performed using R language (version 4.1.2). *p* values of < 0.05 were considered statistically significant. The animal experiment data is displayed as mean ± SD. Statistical analysis was performed using Prism 8 (GraphPad Software). Using one‐way analysis of variance (ANOVA), *p* values of < 0.05 were considered statistically significant.

## 3 Results

### 3.1 Cell Atlas of Early DR

**FIGURE 1:**
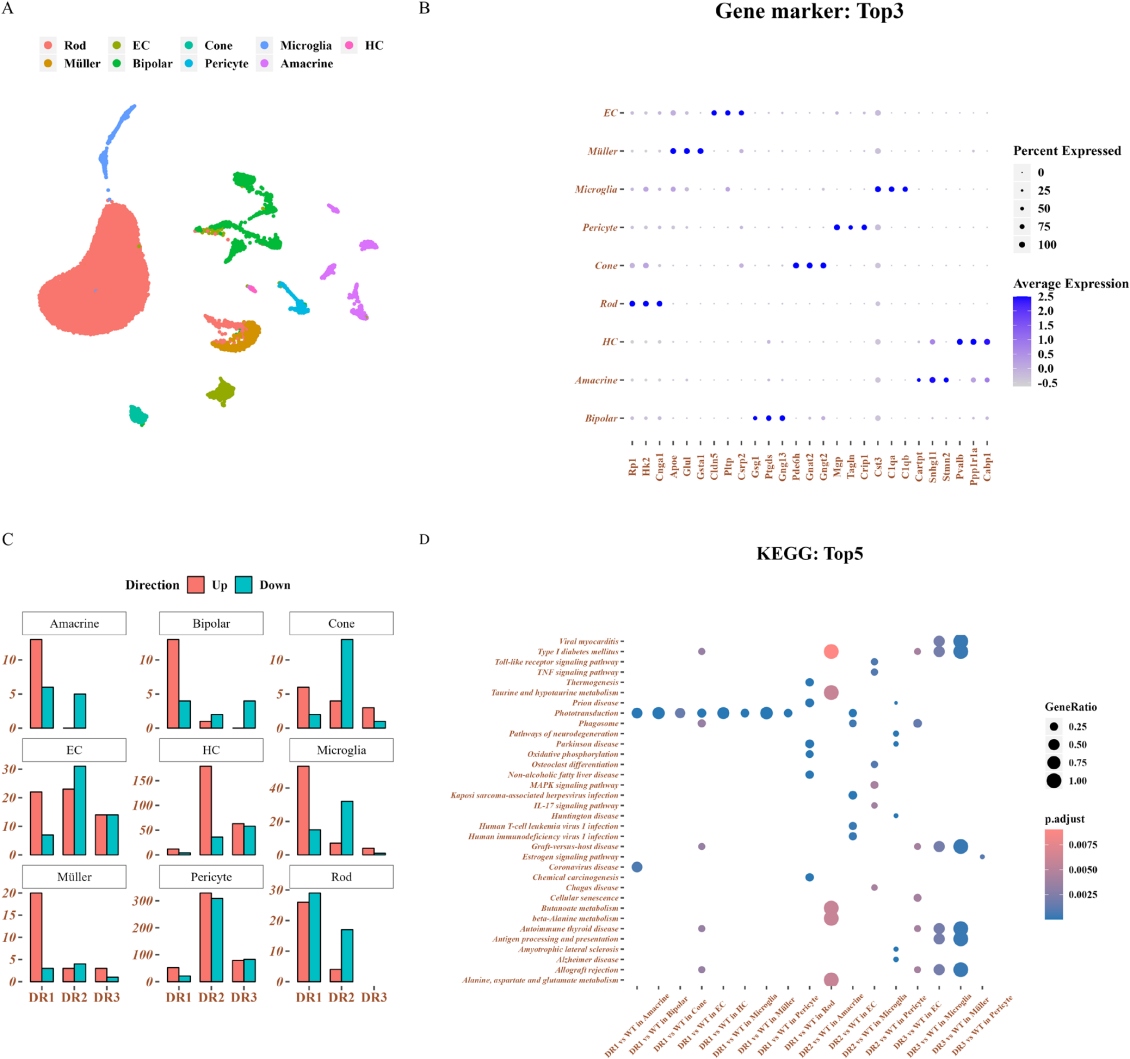
Cell atlas of early DR. (A) UMAP plot displaying different cell types. (B) DotPlot illustrating the top three gene markers of different cell types (the top three multiples of difference). (C) Histogram presenting the statistics of the number of differentially expressed genes (DEGs) in different cell types (all compared to WT). (D) Top three KEGG Enrichment Pathways of Significantly DEGs in Scatter Maps.

A total of 29 cell assemblies were demonstrated using UMAP dimensionality reduction (Figure S1). The 29 cell assemblies were divided into nine cell types, including rod cells, cone cells, horizontal cells (HC), amacrine, bipolar, Müller, Microglia, EC, and pericytes, according to the previously reported list of genes expressed in retinal cell types (Figure 1A) [22]. Rod, cone, HC, amacrine, and bipolar were the nerve cells among the nine cell types, whereas Müller and Microglia were the glial cells. Figure 1B illustrates the top three canonical markers of each cell cluster, such as Rp1, Hk2 and cngal for rod and pde6h, gnat2 and mg for cone. Meanwhile, we analysed and calculated the DEGs between WT and DR according to the nine cell types. Samples in the DR1 group demonstrated the strongest changes of disturbance in amacrine, bipolar, microglia and rod in terms of the number of DEGs. The cone, EC, HC and pericyte with significant changes of disturbance were observed in the DR2 samples. However, a few perturbation genes were determined in DR3 relative to DR1 or DR2 (Figure 1C). Moreover, the results of GSVA indicated that the DEGs in different cell types were all enriched in the phototransduction pathway in the DR1 samples. These results indicated that the early stage of DR may be associated with rhodopsin activation in rods.

### 3.2 Changes in EC in the Early Stage of DR

**FIGURE 2:**
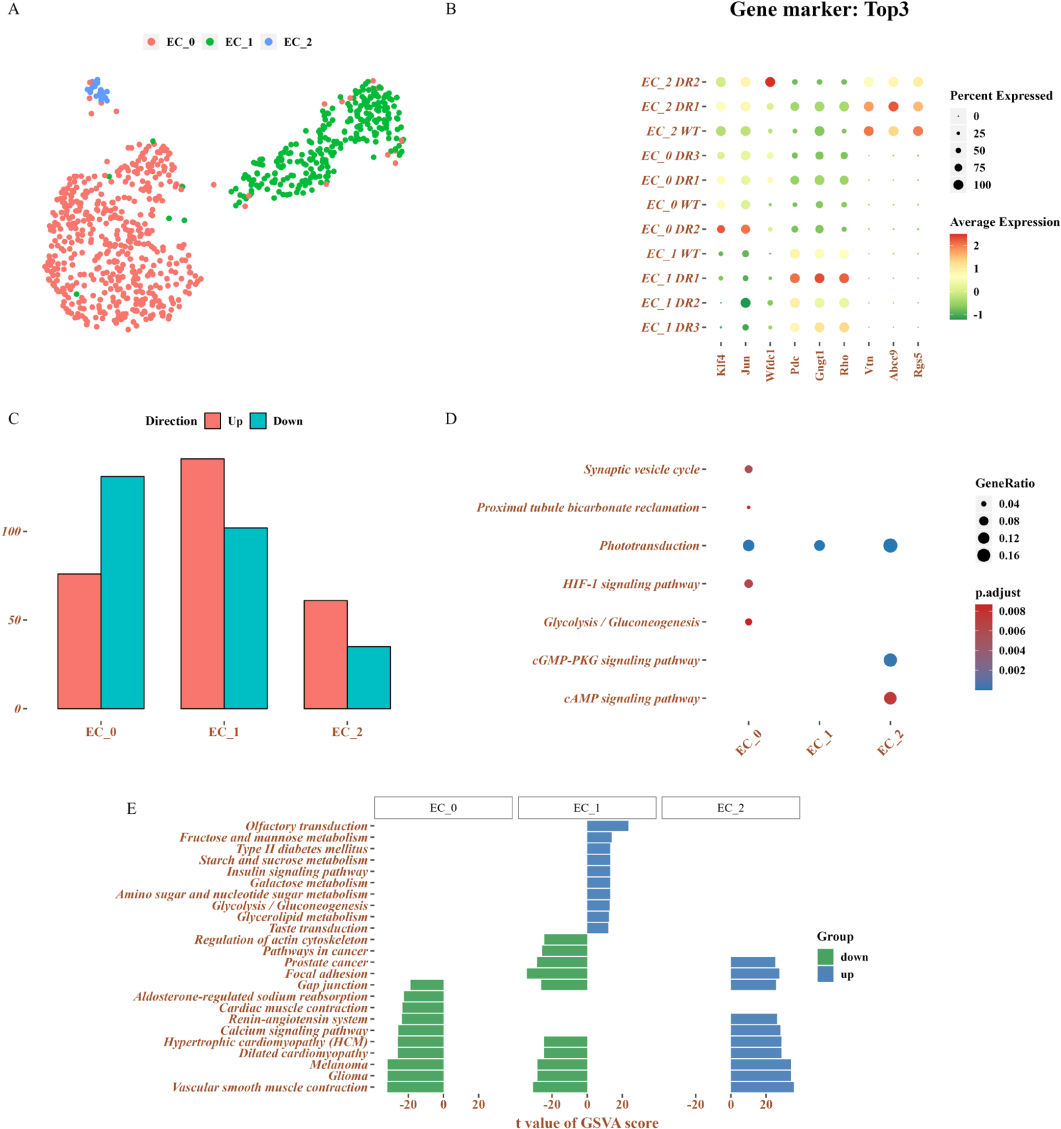
Changes in EC in the early stage of DR. (A) The UMAP diagram shows different EC subtypes. (B) DotPlot displays EC and Pericyte A and pericyte, the top three gene markers of A. (C) The bar chart illustrates EC_0, EC_1, and EC_2, the number of differentially expressed genes. (D) Scatter plot shows significant differences in the expression of genes in the enrichment pathway. (E) Bidirectional histogram illustrates differences in the GSVA enrichment pathway among different subgroups.

Abnormal proliferation of retinal blood vessels is the main characteristic of DR. Thus, investigating the changes in EC could help understand the mechanism of the early stage of DR. We revealed that EC mainly localised in DR1 samples, as visualised in the UMAD dimensionality reduction, indicating that DR in the early stage due to EC proliferation affects or causes EC proliferation. ECs appear to play a protective role in reducing their injury at the early stage. As previously described, we revealed that EC could significantly activate phototransduction, which is a significant source of oxidative stress in diabetic animals [23]. Previous studies revealed that degeneration or blocking phototransduction protects against vascular DR [24, 25]. Hence, the EC was further divided into EC_0 (505 cells), EC_1 (263 cells) and EC_2 (24 cells) to explore the detailed function of EC in DR (Figure 2A). Additionally, the EC subtypes were marked with specific genes (Figure 2B). The GSVA results confirmed that EC_0, EC_1 and EC_2 were enriched in the phototransduction signalling pathway, which is consistent with the previous description. Moreover, we revealed that the DEGs in EC_0 were significantly involved in the synaptic vesicle cycle, proximal tubule bicarbonate reclamation, HIF‐1 signalling pathway and glycolysis/gluconeogenesis (Figure 2C,D). Among these signalling pathways, the HIF‐1 signalling pathway was the key molecular mechanism that inhibited retinal endotheliocyte activation and then suppressed DR occurrence [26].

Furthermore, GSVA enrichment analysis conducted to comprehensively understand and compare the difference in signalling pathways among EC_0, EC_1 and EC_2 revealed that both EC_0 and EC_1 downregulated the vascular smooth muscle contraction signalling pathway, whereas the function of which was improved in EC_2.

### 3.3 Changes in PRCs in the Early Stage of DR

We revealed that PRCs, including rod and cone, were differentially distributed between DR (DR1, DR2 and DR3) and WT from the results of the cell atlas. Thereby, the PRCs were reclustered into eight subclusters (0–7) with a resolution of 0.3 (Figure S3). The results of clustering indicated that Subclusters 1 and _3 were mainly distributed in DR1, Subclusters 2 and 5 in DR2, and subclusters 0 and 4 in DR3 and WT (Figure S4).

**FIGURE 3:**
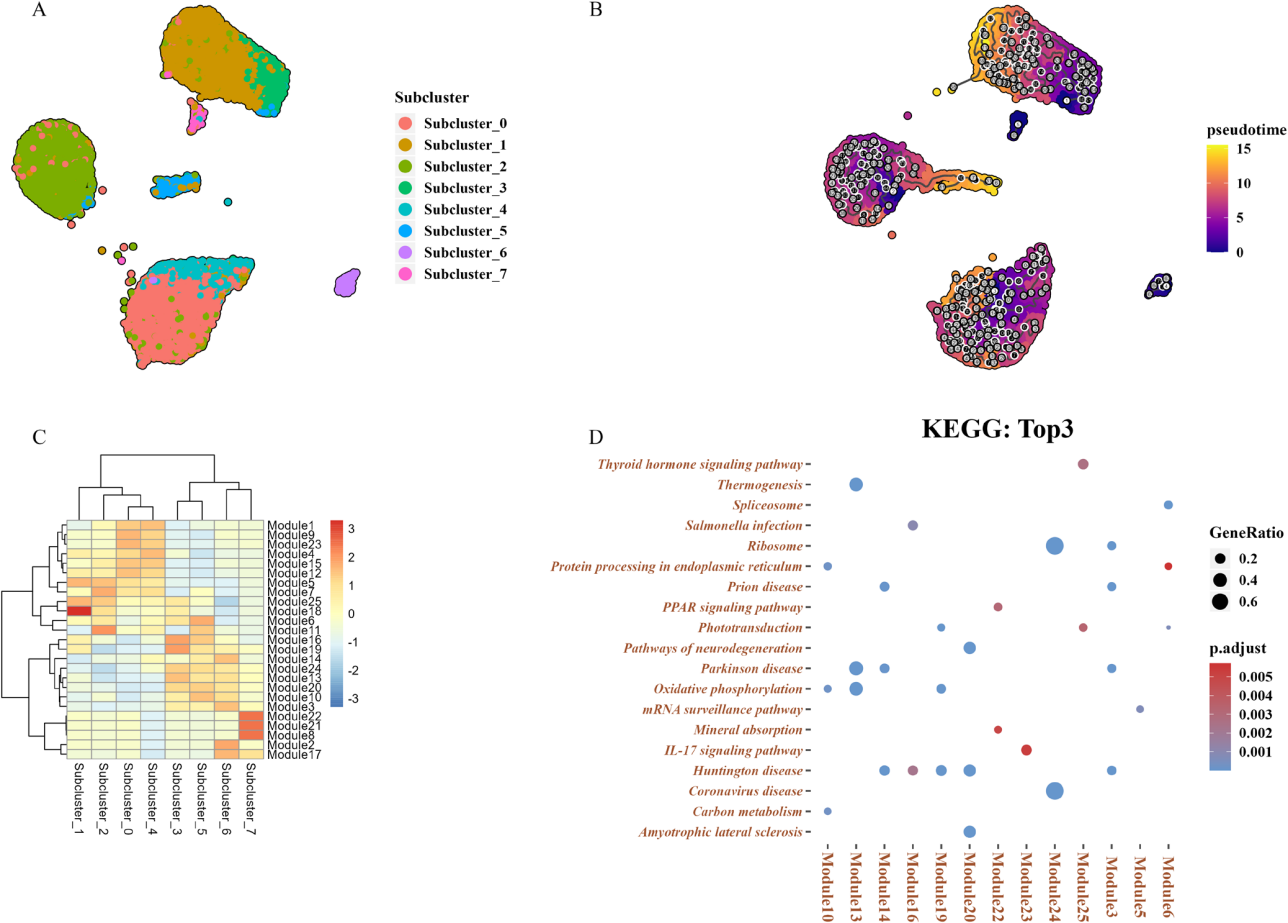
Changes in photoreceptor cells in the early stage in DR. (A) The UMAP image illustrates subcluster (B) of photosensitive cells, whereas the UMAP image presents pseudo time. (C) The association between significantly important gene modules in the heatmap and the subclass. (D) Scatter plot display of KEGG enrichment results of module genes.

Further process trajectory analysis revealed a similar trajectory among different subclusters and was separated into three branches: from Subcluster 3 to Subcluster 1; from Subcluster 2 to Subcluster 5; and from Subcluster 0 to subcluster 4 (Figure 3A,B). Notably, we revealed that the trajectory of Subclusters 3–1 mainly focused on DR1, indicating that the PRCs in the early DR changed from Subclusters 0 and 4 to Subclusters 3 and 1, respectively. Moreover, 3745 differential genes in the trajectory were identified, based on which, 25 coexpression modules were observed with a resolution of 0.01. Correlation analysis on coexpression modules and cell types indicated that trajectory Subcluster 0 was highly related to modules 9 and 23, Subcluster 1 correlated to modules 18 and 25, and Figure 3C illustrates the detailed information.

**FIGURE 4:**
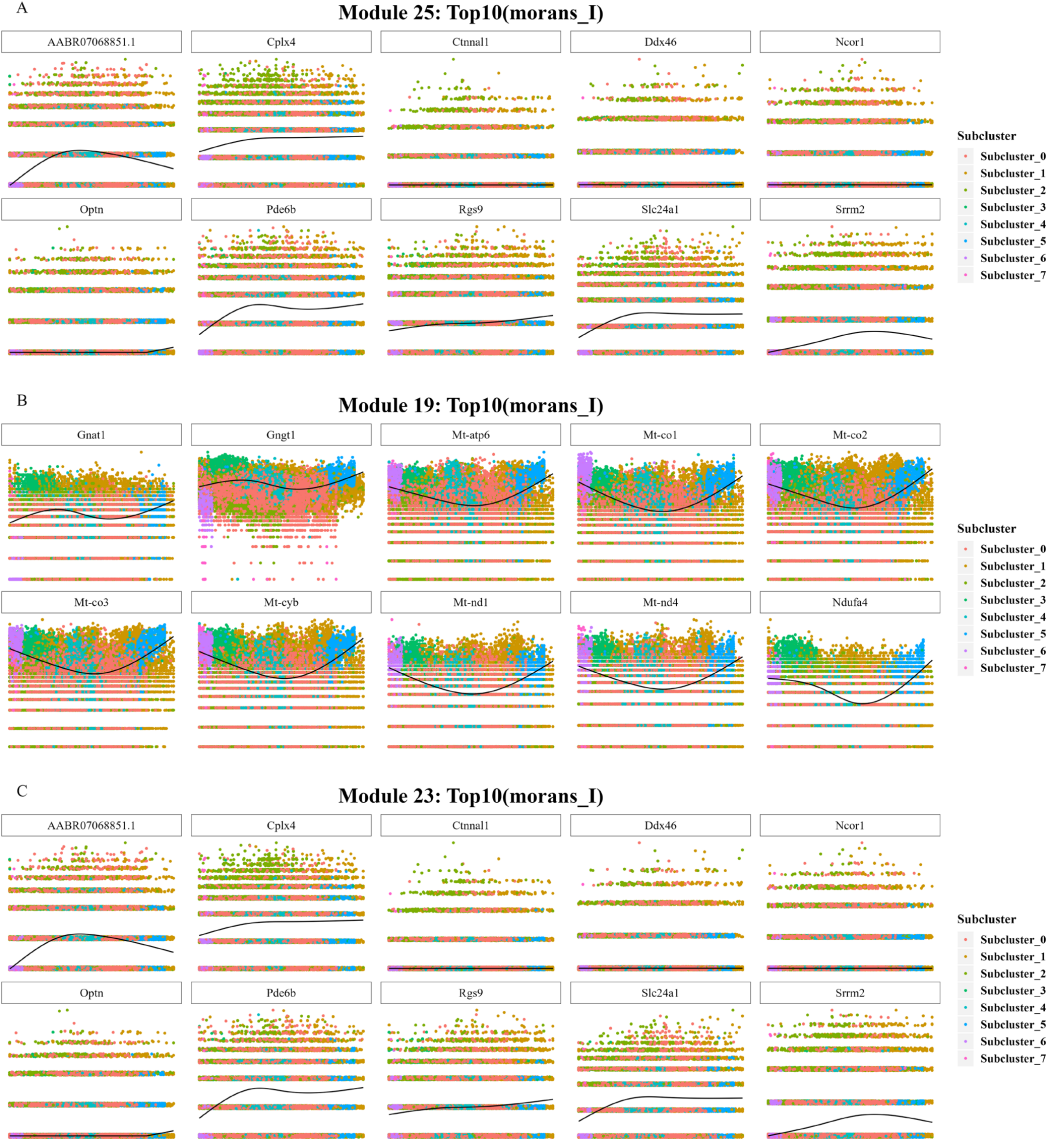
Correlation between modules and pseudotime. (A) Scatter plot showing the association between the module 25 marker gene and the pseudotime (marker gene: Morans_I top 10). (B) Scatter plot illustrating the association between the module 19 marker gene and the pseudotime (marker gene: Morans_I top 10). (C) Scatter plot illustrating the association between the module 23 marker gene and the pseudotime (marker gene: Morans_I top 10).

Moreover, function analysis was conducted on the modules that revealed module 25 associated with Subcluster 1 enriched in the thyroid hormone signalling pathway, which is consistent with the previous study [27]. Module 19 related to Subcluster 3 was involved in phototransduction, which was an important pathway in DR, consistent with the results in Figure 2C. Thus, DEG expressions in module 19 may be the key reason why the patients' version was affected in the early DR. Module 23 related to subcluster 0 participated in the interleukin (IL)‐17 signalling pathway, and Module 6 (Subcluster 5) enriched in protein processing in the endoplasmic reticulum and spliceosome (Figure 3D). We revealed that modules 25 (Subcluster 1), 19 (Subcluster 3) and 23 (Subcluster 0) were relatively important in contrast to the other modules based on the above results. Subclusters 1 and 3 are mainly distributed in DR1, whereas Subcluster 0 is distributed in DR3 and WT. Figure 4 illustrates further correlation analysis on modules 25, 19 and 23.

### 3.4 Changes in Cell Communication Between EC and PRCs in Early DR

**FIGURE 5:**
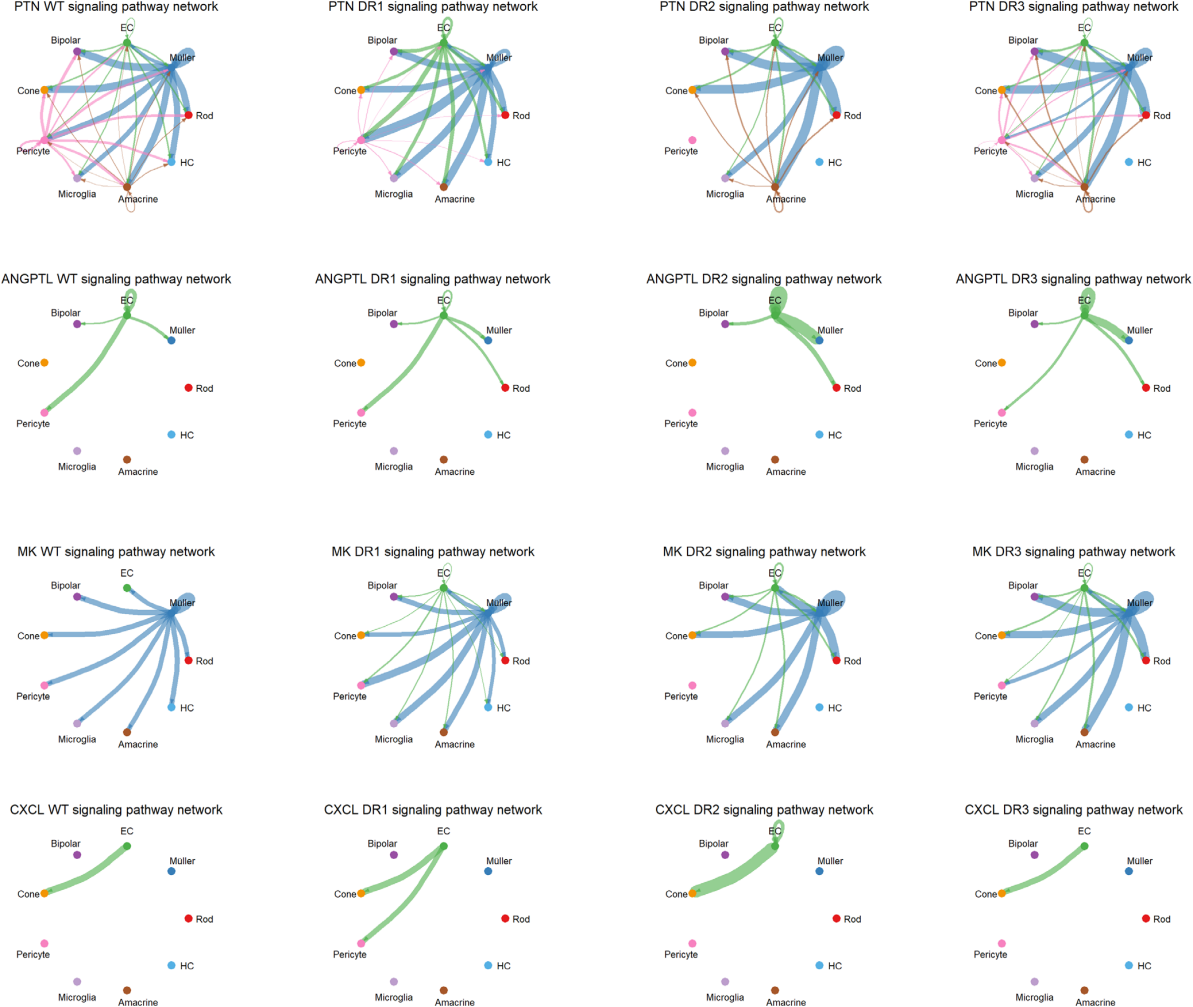
Changes in cell communication between EC and PRCs in early DR. Cellular communication intensity of the PTN, ANGPTL0, MK and ANGPTL signalling pathways in WT, DR1, DR2 and DR3 mice.

**FIGURE 6:**
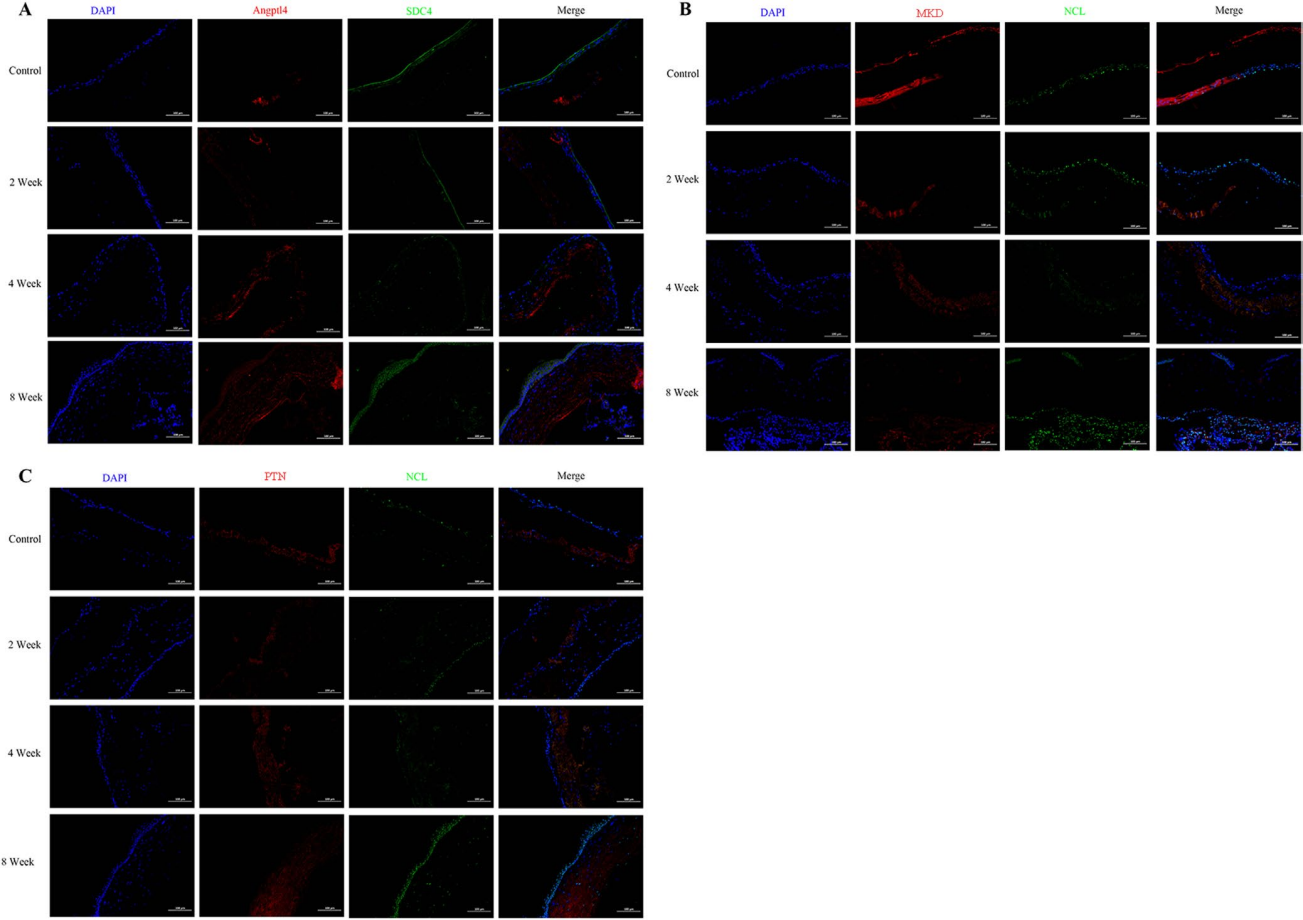
Immunofluorescence verified the expression of ligand–receptor in DR at 2, 4, and 8 weeks. (A–C) The expressions of Ptn‐Ncl, Mkd‐Ncl and Angptl4‐Sdc4 in the retina were detected by immunofluorescence at 2, 4 and 8 weeks after DR Modelling.

Previous results have confirmed the role of ECs and PRCs in early DR. Therefore, we further investigated the interaction between both. Figure S5A–D illustrate that, in terms of outgoing signalling patterns, PRCs (including rod and cone) were regarded as the source of cell communication. The rod cells, as targets, received information from the PTN, MK and ANGPTL signalling pathways from the perspective of the incoming signalling patterns. The cone cells received information from the PTN, MK and CXCL signalling pathways (Figure S5E–H). Therefore, the carrier of the signal communication between ECs and PRCs should be locked in the signalling pathways of PTN, MK and ANGPTL in this study. The PTN signalling pathway, among the three signalling pathways, is involved in signal communication between ECs and PRCs in the DR1 sample, and its intensity exceeds that of WT, DR2 and DR3. Early DR may cause EC proliferation and further affect PRCs. Both the ANGPTL and MK signalling pathways only participate in signal communication between ECs and PRCs in DR1, DR2 and DR3, indicating that DR may activate the two pathways (Figure 5). Meanwhile, detailed pairs of source –target were significantly changed in cell communication between ECs and PRCs, including Ptn‐Ncl, Mkd‐Ncl and Angptl4‐Sdc4. Furthermore, the results of immunofluorescence staining indicated that the expression of Ptn‐Ncl, Mkd‐Ncl and Angptl4‐Sdc4 was upregulated in DR and reached the highest level in DR at 8 weeks (Figure 6).

### 3.5 AAV‐shANGPTL4 Alleviates Retinal Dysfunction and the Level of Ligand–Receptor in DR Rats

**FIGURE 7:**
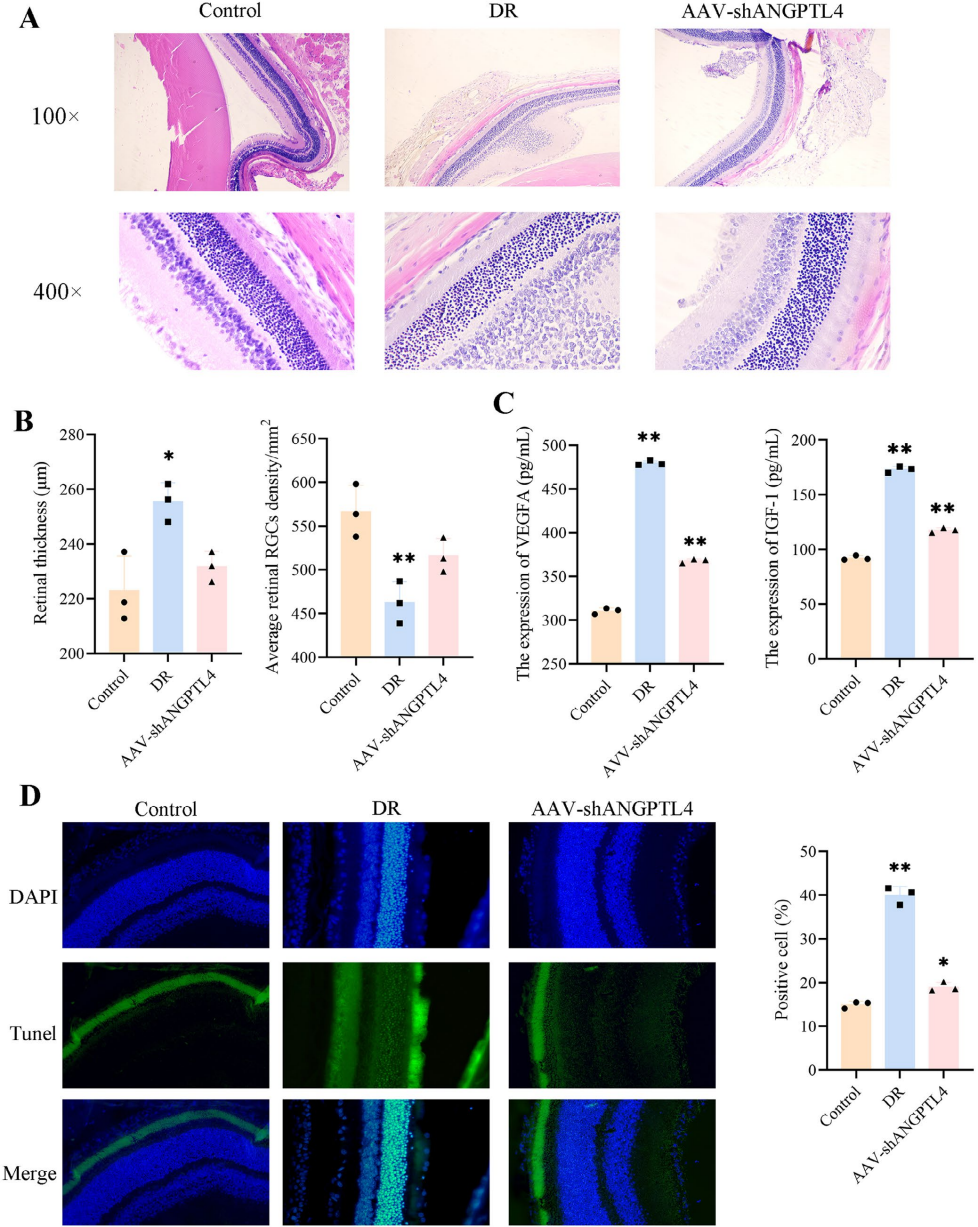
AAV‐shANGPTL4 alleviates retinal dysfunction in vivo. (A and B) Retinal HE images of DR rats; Retinal thickness and mean density of retinal ganglion cells were measured; (C) The expression levels of VEGFA and IGF‐1 in the serum of rats were detected by ELISA; (D) TUNEL staining was used to detect retinal apoptosis and Statistical analysis (400×). Above panel: DAPI; Middle panel: FITC; Below panel: Merged (DAPI and FITC), *p < 0.05 and **p < 0.01.

Through the analysis of single‐cell sequencing data, we found that ANGPTL4 may be a potential therapeutic target for DR, so we constructed AAV‐shANGPTL4 for DR Treatment. The results showed that no obvious retinopathy occurred in the control group, while the retina of the model group was obviously thickened, with blurred layers and chaotic arrangement. Compared with the model group, the retinopathy of rats in the AAV‐shANGPTL4 group was alleviated, the retinal thickness was significantly reduced, and the average density of RGCs was improved (Figure 7A,B). These results indicate that the degree of retinopathy can be improved after ANGPTL4 knockdown.

We detected the expression levels of VEGFA and IGF‐1 in the serum of rats in each group by ELISA kit. The results showed that the expressions of VEGFA and IGF‐1 in the model group were significantly upregulated, while those in the AAV‐ANGPTL4 group were improved (Figure 7C). At the same time, TUNEL staining was also performed on the retinal tissues of rats to observe the apoptosis of retinal tissues, and it was found that the AAV‐ANGPTL4 group could also improve the upregulation of the apoptosis rate caused by the occurrence of retinopathy (Figure 7D). These results indicated that the AAV‐ANGPTL4 group could significantly reduce the angiogenesis of diabetic retinopathy and promote the apoptosis of neonatal retinal endothelial cells.

**FIGURE 8:**
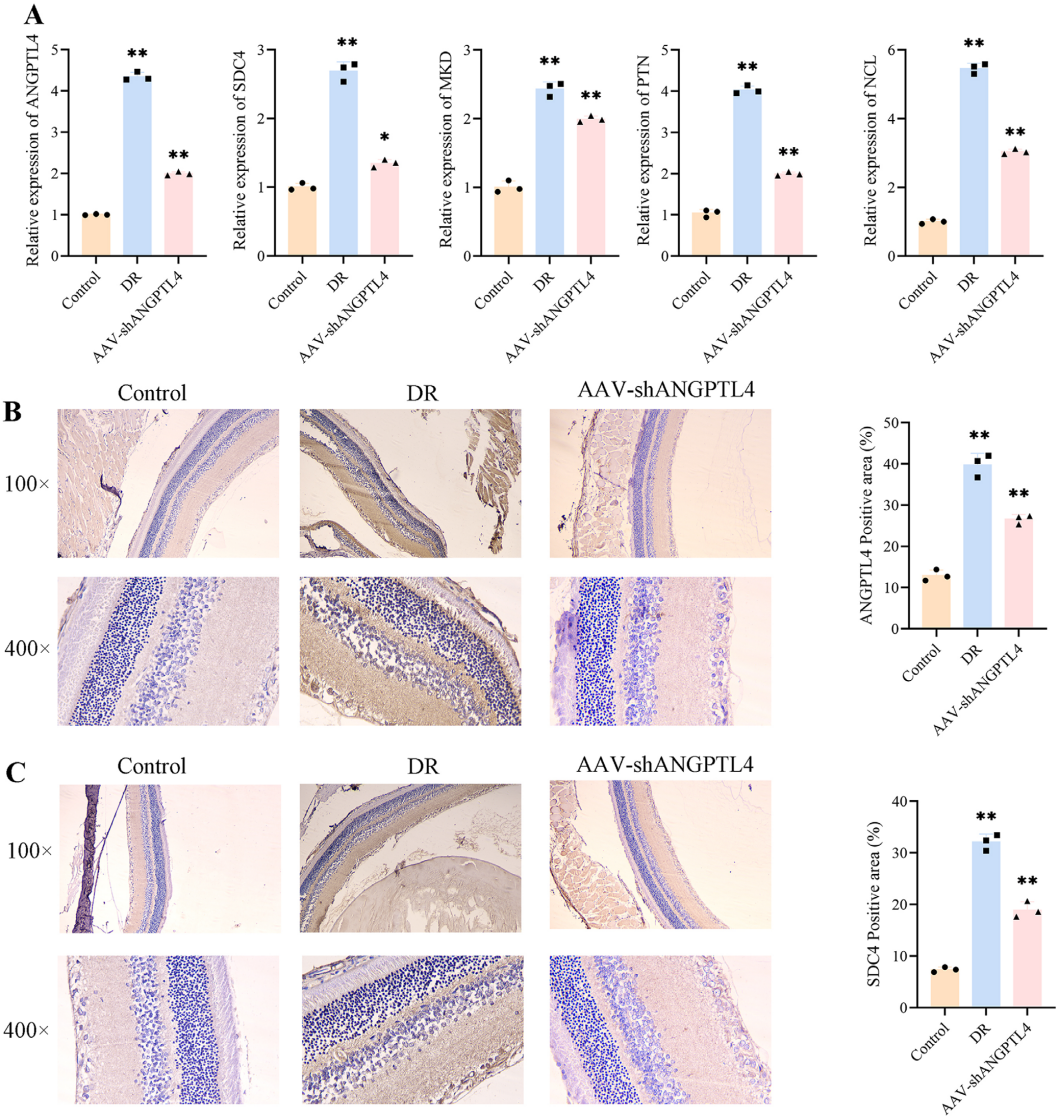
AAV‐shANGPTL4 improves the level of ligand receptor in DR rats. (A) QPCR was used to detect the SD rat retinal mRNA levels of ANGPTL4, SDC4, MKD, PTN, and NCL in control, model, and AAV‐shANGPTL4 at 8 weeks. Compared with the control group, (B and C) immunohistochemical tests, the positive area was the expression of SDC4 and ANGPTL4. *p < 0.05 and **p < 0.01.

Next, mRNA expression levels of ANGPTL4, SDC4, MKD, PTN, and NCL were detected by qPCR, and the results were consistent with the results of single‐cell sequencing analysis and immunofluorescence. When DR Lasted for 8 weeks, the expressions of ANGPTL4, SDC4, MKD, PTN, and NCL were significantly increased. The expression of AAV‐shANGPTL4 decreased after treatment. The results of the analysis were verified, and it was further demonstrated that ANGPTL4 could improve the expression level of ligand‐receptor while reducing retinopathy (Figure 8A). The positive areas of ANGPTL4 and SDC4 in the model group were significantly upregulated compared with the control group in protein level. The expression of ANGPTL4 and SDC4 decreased after treatment with AAV‐shANGPTL4 (Figure 8B,C).

## 4 Discussion

Popularly, the iBRB plays a crucial role in the pathogenesis of various retinal diseases and is a vital structure that ensures retinal homeostasis. Exploring the mechanisms of the destruction and steady state of the iBRB would help improve DR treatment. Müller cells, ECs, and pericytes have been considered important components of the iBRB. Wang et al. revealed the importance of the Müller cells and pericytes in the early stage of DR [22]. However, the role of ECs in the iBRB remains unclear. Moreover, recent reports indicated a specialised neural cell type, PRCs, in DR development [18], which was the leading cause of vision loss. Thus, investigating the role of ECs and PRCs in DR may help understand the mechanism of vision loss caused by DR.

ECs line the blood vessels in the retina and play a crucial role in maintaining the integrity and function of the iBRB [8]. ECs in the retinal blood vessels in DR become damaged and dysfunctional due to the chronic hyperglycaemia related to diabetes [9]. Hence, the ECs undergo various pathological changes, including tight junction protein loss, increased vascular permeability and neovascularisation [28, 29]. Consistently, this study revealed that ECs are involved in the HIF‐1 and vascular smooth muscle contraction signalling pathways. Previous studies have demonstrated that these pathways were the key molecular mechanism to inhibit iBRB damage and then suppress DR occurrence [30, 31, 32]. Furthermore, PRCs are specialised cells in the retina that detect and convert light into electrical signals, which are then transmitted to the brain for visual processing. PRCs are significantly affected in DR due to the compromised iBRB although not a direct part of the iBRB. Meanwhile, we revealed that PRCs participated in the IL‐17 and PPAR signalling pathways that were related to inflammatory reactions [21, 33, 34]. Above these descriptions, ECs in the retinal blood vessels maintain iBRB integrity, and their dysfunction in the DR contributes to increased vascular permeability and neovascularisation [15]. The compromised iBRB, in turn, affects the PRCs, causing ischaemia, hypoxia and neural degeneration, resulting in vision loss [35].

Furthermore, vascular ECs and PRCs both play a crucial role in maintaining the integrity and function of the BRB in the context of DR [36]. The connection between vascular ECs and PRCs depends on the proper function of photoreceptors based on a healthy blood supply. The ECs maintain the blood flow and provide essential substances to support the metabolic requirements of the PRCs [37]. Conversely, the function and survival of vascular ECs are influenced by signals from the PRCs and the surrounding retinal cells. BRB disruption causes a vicious cycle of impaired vascular function and photoreceptor degeneration in DR [14]. In our study, the Ptn‐Ncl, Mkd‐Ncl and Angptl4‐Sdc4 pairs of the source‐target were significantly changed in the cell communication between ECs and PRCs.

Among these source targets, angiopoietin‐like protein 4 (Angptl4) is involved in regulating angiogenesis, defined as new blood vessel formation [38]. An imbalance in angiogenesis is observed in DR, causing abnormal blood vessel growth and leakage [39]. Angptl4 is upregulated in response to high glucose levels, inflammation and oxidative stress, which are all implicated in DR. The increased Angptl4 levels in the retina can promote abnormal blood vessel growth by stimulating EC proliferation and migration, which make up the inner lining of blood vessels [40]. This excessive angiogenesis causes leaky blood vessel formation and diabetic macular edema development, which is a predominant complication of DR [41]. Our study further validated that Angptl4 was improved at the early stage of DR. Overall, Angptl4 plays a pathogenic role in DR by promoting abnormal angiogenesis and compromising the blood‐retinal barrier, making it a potential therapeutic target for DR treatment. To explore the mechanism of further ANGPTL4 in DR. Intravitreous injection of AAV‐shANGPTL4 in the DR model, we found that knocking down ANGPTL4 in the retina of diabetic rats can significantly improve the degree of retinopathy and improve the apoptosis of retinal cells. At the same time, the expression of VEGF and IGF‐1 was decreased. VEGFA and its signalling pathway can induce vascular permeability and induce angiogenesis by promoting endothelial cell survival and proliferation [42]. IGF‐1 is involved in the regulation and growth of blood vessels, is the main reason for the growth and proliferation of vascular endothelial cells and is involved in the activation of VEGF, which plays an important role in the pathogenesis of DR [43]. These results suggest that ANGPTL4 reduction can reduce retinal thickness and angiogenesis of DR By reducing the levels of VEGFA and IGF‐1. We also found that the mRNA and protein levels of SDC4 decreased significantly after ANGPTL4 was knocked down. In addition, ANGPTL4 can also improve the mRNA levels of newly obtained Ptn‐Ncl and Mkd‐Ncl. SDC4 is critical in angiogenesis, while SDC4 is upregulated in retinal pathologic angiogenesis and promotes VEGFA to play a role [44]. It confirmed the role of Angptl4‐Sdc4 in DR and the therapeutic effect of ANGPTL4.

## 5 Conclusions

In summary, the ECs and PRCs work together to maintain BRB integrity and function. Dysfunction or damage to either cell type contributes to DR development and progression.

ANGPTL4 can regulate the angiogenesis mechanism of retinal vascular endothelial cells and delay the progression of diabetic retinopathy.

## Author Contributions

**Ning Wang:** conceptualization (equal), data curation (equal), methodology (equal), writing – original draft (equal). **Huibo Li:** conceptualization (equal), methodology (equal), writing – original draft (equal). **Qinqin Sun:** methodology (equal), writing – original draft (equal). **Xuelian Han:** investigation (equal), methodology (equal). **Sheng Su:** conceptualization (equal), data curation (equal), methodology (equal), writing – original draft (equal).

## Ethics Statement

Each animal experimental procedure gained approval from the Animal Ethics Committee of the First Affiliated Hospital of Harbin Medical University.

## Consent

The authors have nothing to report. Consent to Participate: The experimental protocol was performed in accordance with the relevant guidelines and regulations of the Basel Declaration. The study is reported in accordance with ARRIVE guidelines (https://arriveguidelines.org).

## Conflicts of Interest

The authors declare no conflicts of interest.

## Supporting information


**Figure S1.** A total of 29 cell assemblies were obtained from DR.

**Figure S2.** ECs were further divided into EC_0 (505 cells), EC_1 (263 cells) and EC_2 (24 cells).

**Figure S3.** The PRCs were reclustered into eight subclusters (0–7) with a resolution of 0.3.

**Figure S4.** The distribution of the eight subclusters between DR (DR1, DR2 and DR3) and WT.

**Figure S5.** Outgoing and incoming signalling patterns of cell communication between DR (DR1, DR2 and DR3) and WT.

## Data Availability

The data sets used and/or analysed during the current study are available from the corresponding author via email request.
