# Supplementary figures and images for: The Exploration of Disturbance of Capillary and Photoreceptor Communication Networks in Diabetic Retinopathy Through Single‐Cell RNA‐Seq
Source: J Cell Mol Med. 2025 Mar 3;29(5):e70442. doi: 10.1111/jcmm.70442 (PMC11875770; doi:10.1111/jcmm.70442)

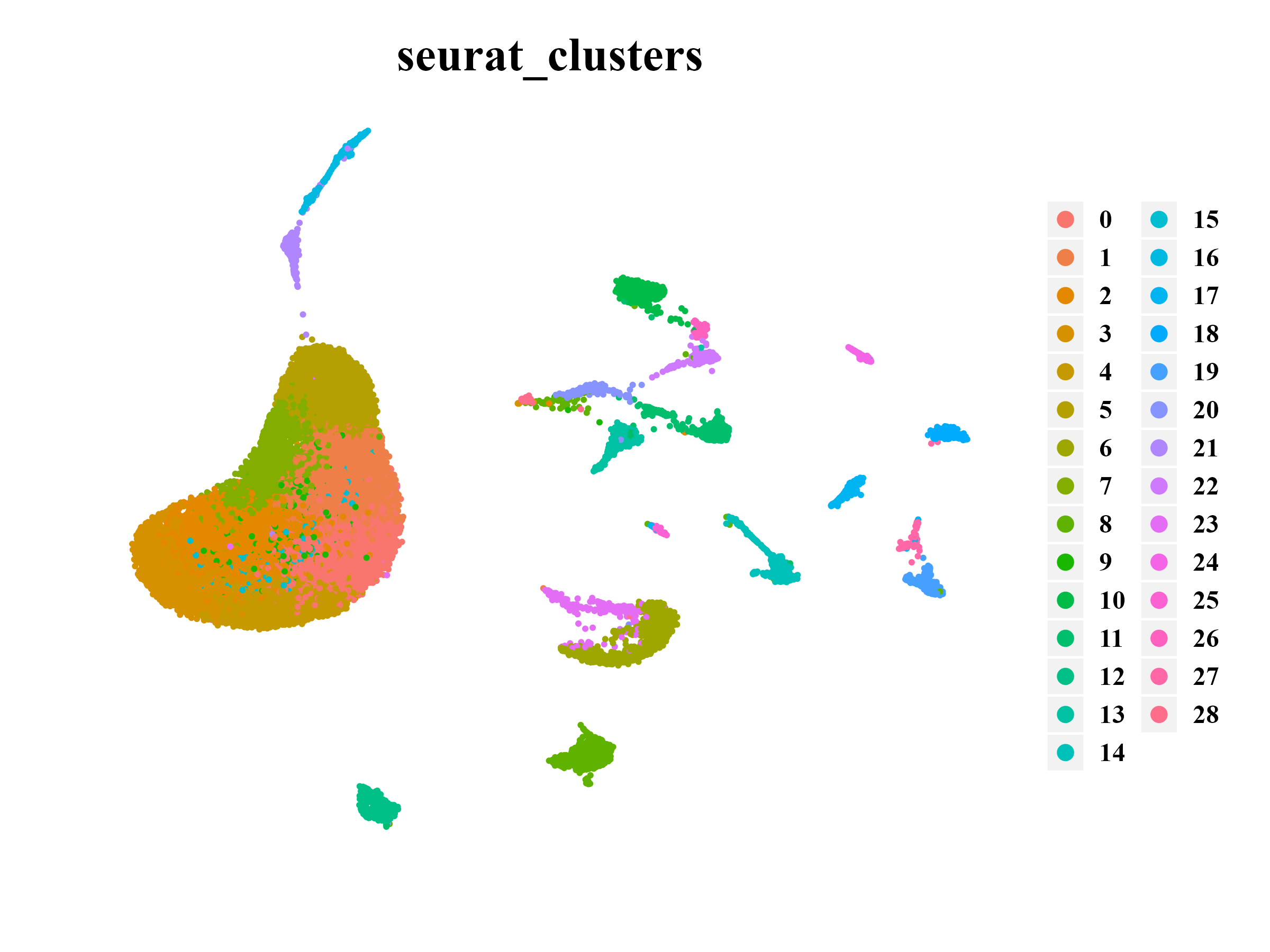

Supplement: Supplementary file 1 — Figure S1. A total of 29 cell assemblies were obtained from DR. [file JCMM-29-e70442-s003.png]

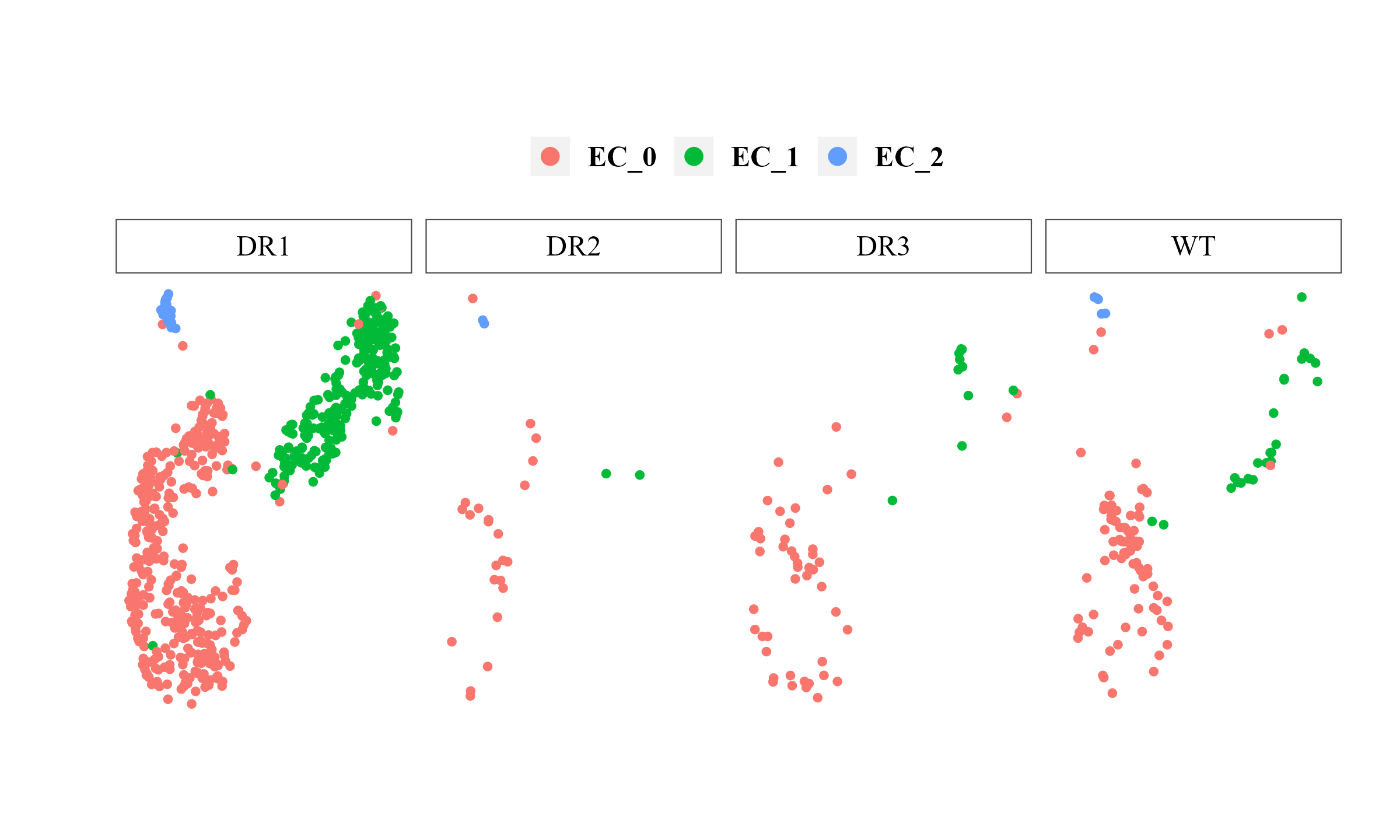

Supplement: Supplementary file 2 — Figure S2. ECs were further divided into EC_0 (505 cells), EC_1 (263 cells) and EC_2 (24 cells). [file JCMM-29-e70442-s004.png]

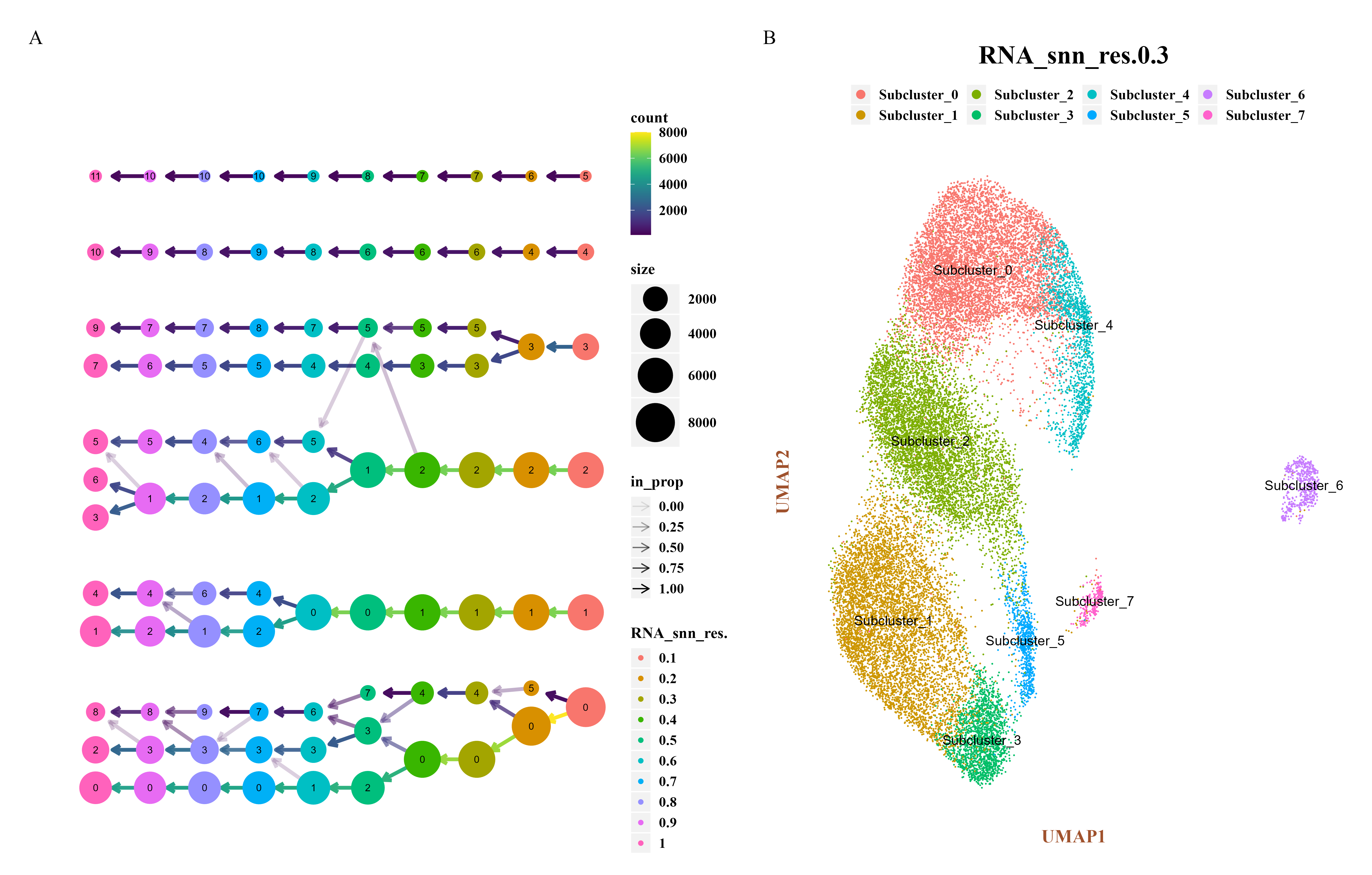

Supplement: Supplementary file 3 — Figure S3. The PRCs were reclustered into eight subclusters (0–7) with a resolution of 0.3. [file JCMM-29-e70442-s001.png]

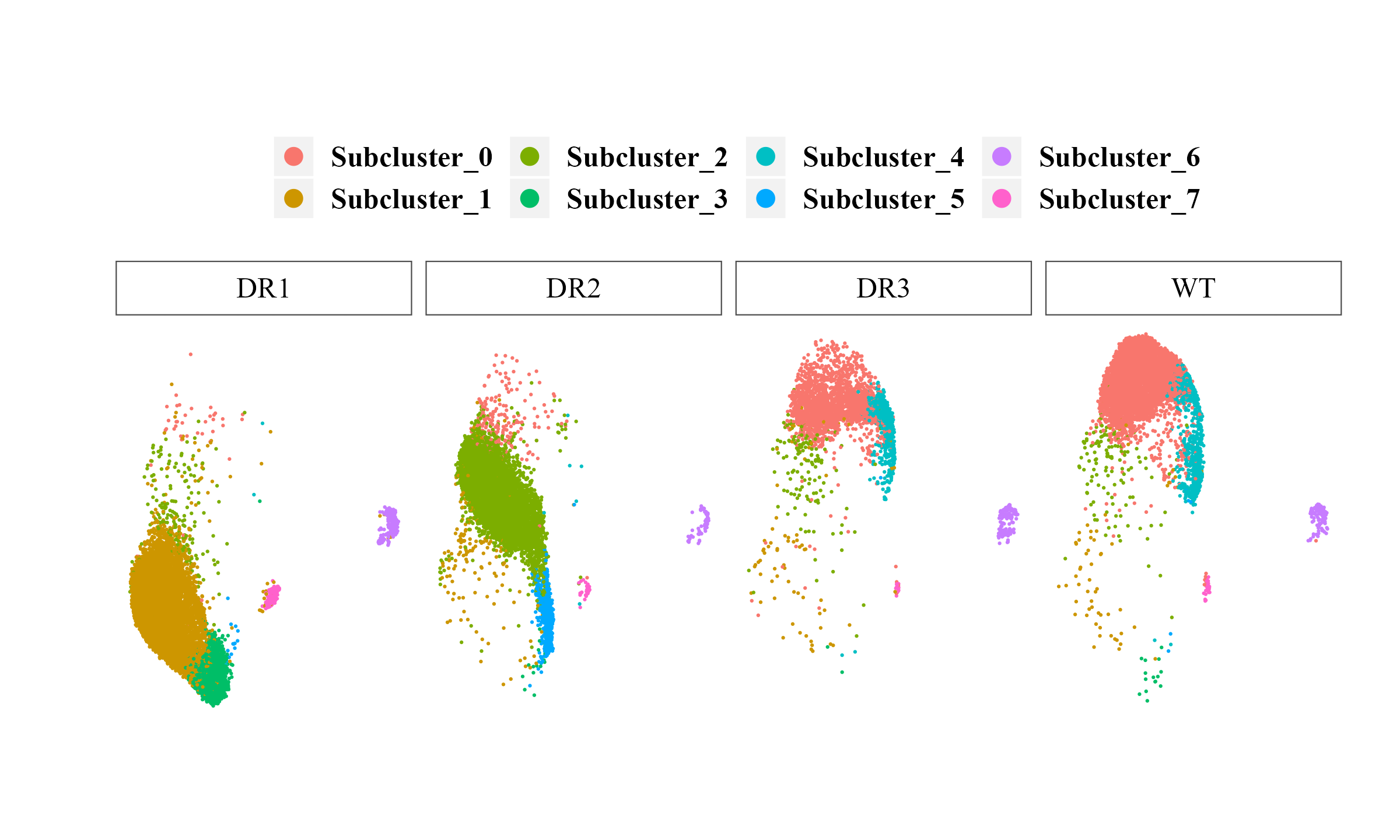

Supplement: Supplementary file 4 — Figure S4. The distribution of the eight subclusters between DR (DR1, DR2 and DR3) and WT. [file JCMM-29-e70442-s005.png]

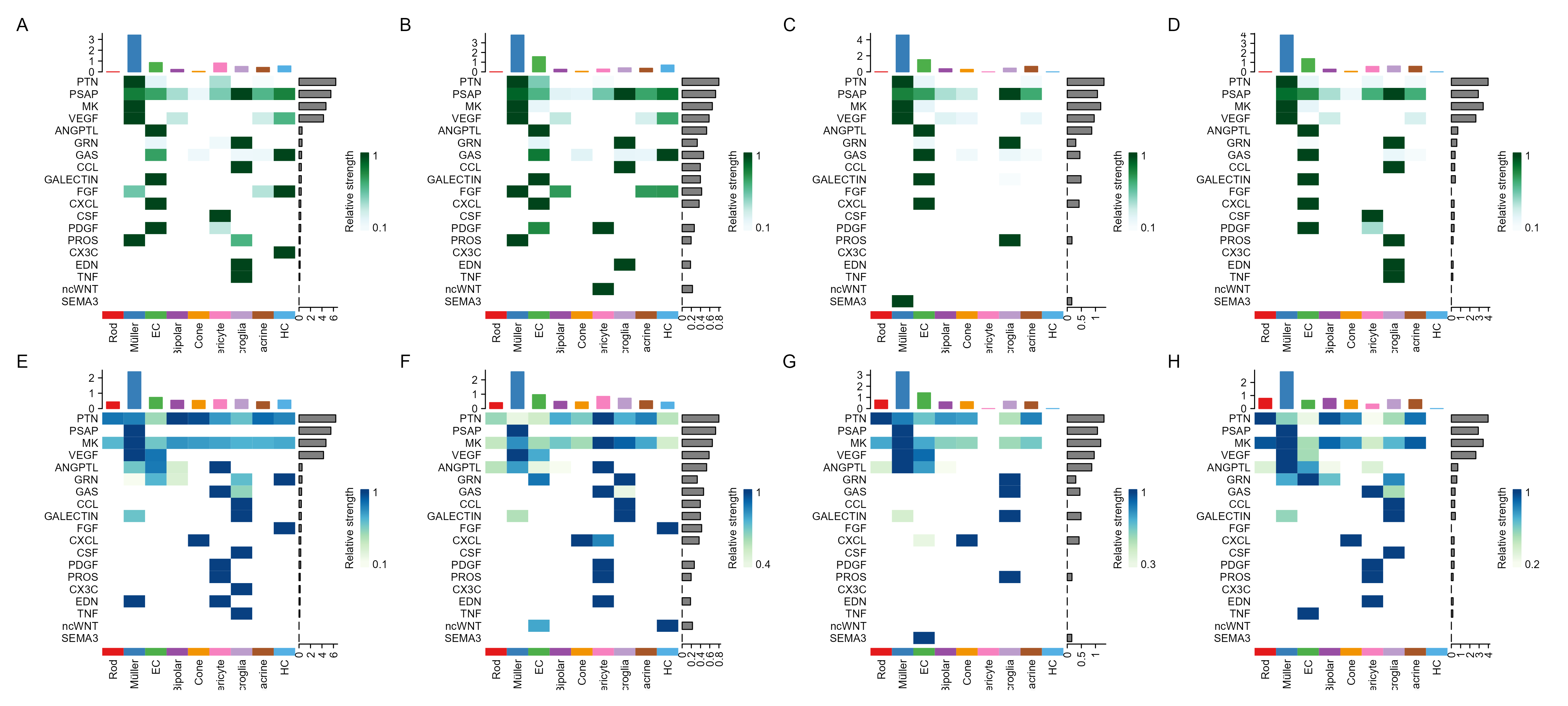

Supplement: Supplementary file 5 — Figure S5. Outgoing and incoming signalling patterns of cell communication between DR (DR1, DR2 and DR3) and WT. [file JCMM-29-e70442-s002.png]
